# Alkaloids from *Veratrum taliense* Exert Cardiovascular Toxic Effects via Cardiac Sodium Channel Subtype 1.5

**DOI:** 10.3390/toxins8010012

**Published:** 2015-12-30

**Authors:** Gan Wang, Ming-Qiang Rong, Qiong Li, Ya-Ping Liu, Cheng-Bo Long, Ping Meng, Hui-Ming Yao, Ren Lai, Xiao-Dong Luo

**Affiliations:** 1 College of Life Sciences, Nanjing Agricultural University, Nanjing 210095, Jiangsu, China; wang.gan@outlook.com (G.W.); 2013116051@njau.edu.cn (H.-M.Y.); 2 Key Laboratory of Animal Models and Human Disease Mechanisms, Chinese Academy of Sciences & Yunnan Province, Kunming Institute of Zoology, Kunming 650223, Yunnan, China; rongmingqiang@mail.kiz.ac.cn (M.-Q.R.); longchb@163.com (C.-B.L.); pingmeng@yeah.net (P.M.); 3 State Key Laboratory of Phytochemistry and Plant Resources in West China, Kunming Institute of Botany, Chinese Academy of Sciences, Kunming 650201, Yunnan, China; qiongqiongbird@163.com (Q.L.); liuyaping@mail.kib.ac.cn (Y.-P.L.)

**Keywords:** *Veratrum taliense*, Na_V_1.5, cardiovascular toxicity

## Abstract

Several species of the genus *Veratrum* that produce steroid alkaloids are commonly used to treat pain and hypertension in China and Europe. However, *Veratrum* alkaloids (VAs) induce serious cardiovascular toxicity. In China, *Veratrum* treatment often leads to many side effects and even causes the death of patients, but the pathophysiological mechanisms under these adverse effects are not clear. Here, two solanidine-type VAs (isorubijervine and rubijervine) isolated from *Veratrum taliense* exhibited strong cardiovascular toxicity. A pathophysiological study indicated that these VAs blocked sodium channels Na_V_1.3–1.5 and exhibited the strongest ability to inhibit Na_V_1.5, which is specifically expressed in cardiac tissue and plays an essential role in cardiac physiological function. This result reveals that VAs exert their cardiovascular toxicity via the Na_V_1.5 channel. The effects of VAs on Na_V_1.3 and Na_V_1.4 may be related to their analgesic effect and skeletal muscle toxicity, respectively.

## 1. Introduction

Several species of the *Veratrum* genus, such as *V. album*, *V. californicum*, *V. viride* and *V. nigrum*, are poisonous to humans and animals, and the principal toxic components are steroid alkaloids. More than 200 different alkaloids belonging to seven groups have been identified from the *Veratrum* species [1,2]. Humans and animals exhibit toxic symptoms after the ingestion of *Veratrum* alkaloids, including vomiting and abdominal pain, followed by cardiovascular disorders, such as bradycardia, hypotension, cardiac arrhythmias and death. Even more, two patients died after using *V. taliense* alkaloids to treat schizophrenia due to the necrosis of cardiomyocytes. Many studies have investigated the possible mechanisms of *Veratrum* alkaloid (VA) toxicities, but only a few works focus on the ion channel. The effects of the VA veratridine on Na current behavior were first detected on neuroblastoma cells using single-channel and whole-cell voltage-clamp recordings [3]. Veratridine modified and opened sodium channels, causing sodium channels to remain open during a sustained membrane depolarization by abolishing inactivation [4]. Another study in guinea pig ventricular myocytes indicated that veratridine modified two different types of single sodium channels (high and low conductance) in cardiac myocytes [5]. Furthermore, batrachotoxin (BTX) was used to demonstrate the binding site of veratridine in the S6 segment of domain D2 (central pore region) of voltage-gated sodium channels (VGSCs) [6]. These studies revealed that VAs interacted with sodium channels and cause the persistent inflow of Na^+^. Now, veratridine is used as an activator of sodium channels and a tool to investigate the function of other chemical compounds [7]. However, the identities of sodium channel subtypes that are the targets of VAs are not known.

The diversity of sodium channel subtypes and functions necessitates the identification of selective sodium channel targets of VAs to understand the pharmacological and side effect profiles of VAs. This study investigated the cardiovascular toxicities of two VAs (isorubijervine and rubijervine) and their interactions with sodium channel subtypes.

## 2. Results

### 2.1. Acute Toxicity of Isorubijervine and Rubijervine on Mice

**Figure 1:**
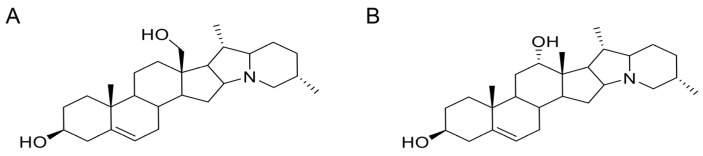
Structures of two toxic alkaloids. (**A**) Isorubijervine; (**B**) rubijervine.

The lethal toxicities of isorubijervine and rubijervine (Figure 1) following tail vein injections were investigated in mice (Table 1). Isorubijervine and rubijervine are highly toxic compounds with LD_50_ (50% lethal dose) of 1.14 and 1.77 mg/kg in mice, respectively.

**Table 1:** Groups of toxic reaction and animal deaths.

Compounds	Total Number of Mice ^b^	Drug Concentration (mg/kg)	Number of Toxic Symptoms	Number Dead	LD_50_ (mg/kg)
Solvent control ^a^	5	-	0	0	-
Isorubijervine	5	2	5	5	1.14 ± 0.10
	10	1.5	10	8	
	10	1	10	4	
	10	0.75	4	0	
	10	0.5	0	0	
Rubijervine	5	7	5	5	1.77 ± 0.20
	10	5	10	8	
	10	2	10	6	
	10	1.5	10	2	
	10	1	9	0	
	10	0.5	1	0	

^a^ Menstruum is 40% DMSO in normal saline. ^b^ All mice were administered a single dose of the drug in the same volume (100 µL) via tail vein injection.

### 2.2. Effects of Isorubijervine and Rubijervine on Rat ECG

ECG signals were recorded using a three-lead surface ECG. Surface ECG parameters in sedated animals were significantly different between treated and control rats (Table 2).

**Table 2:** Surface ECG parameters in sedated control and treated rats. Values are given as the mean ± SE, *n* = 3.

ECG Parameters	Control	Isorubijervine	Rubijervine
Heart rate (beats/min)	371.75 ± 4.65	114.73 ± 13.05	160.23 ± 5.32
P wave duration (ms)	15.81 ± 0.96	19.48 ± 0.41	20.03 ± 0.25
QRS wave duration (ms)	9.83 ± 0.41	9.90 ± 0.11	9.87 ± 0.21
PQ interval (ms)	33.72 ± 0.81	104.05 ± 2.98	120.54 ± 1.83
QT interval (ms)	29.42 ± 0.66	25.33 ± 0.55	25.67 ± 0.43
QRS amplitude (mV)	0.32 ± 0.01	0.50 ± 0.01	0.41 ± 0.01
P amplitude (mV)	0.035 ± 0.004	0.037 ± 0.004	0.037 ± 0.004

Control rats exhibited a heart rate of 371.75 ± 4.65 beats/min. The heart rates in 1 mg/kg isorubijervine- and rubijervine-treated rats were 114.73 ± 13.05 and 160.23 ± 5.32 beats/min, respectively. The P wave duration in control rats was 15.81 ± 0.96 ms. The P wave duration was significantly prolonged in rats treated with isorubijervine (19.48 ± 0.41 ms) or rubijervine (20.03 ± 0.25 ms). These compounds did not affect QRS wave duration nor the amplitude of P waves in rats. However, the PQ interval of the VA treatment groups increased significantly from 33.72 ± 0.81 ms to 104.05 ± 2.98 and 120.54 ± 1.83 ms after treatment with isorubijervine and rubijervine, respectively. The QT interval (29.42 ± 0.66 ms) in control rats was shortened to 25.33 ± 0.55 and 25.67 ± 0.43 ms by isorubijervine and rubijervine treatment, respectively. Isorubijervine or rubijervine administration increased the QRS amplitude from 0.32 ± 0.01 mV in control rats to 0.50 ± 0.01 and 0.41 ± 0.01 mV, respectively.

**Figure 2:**
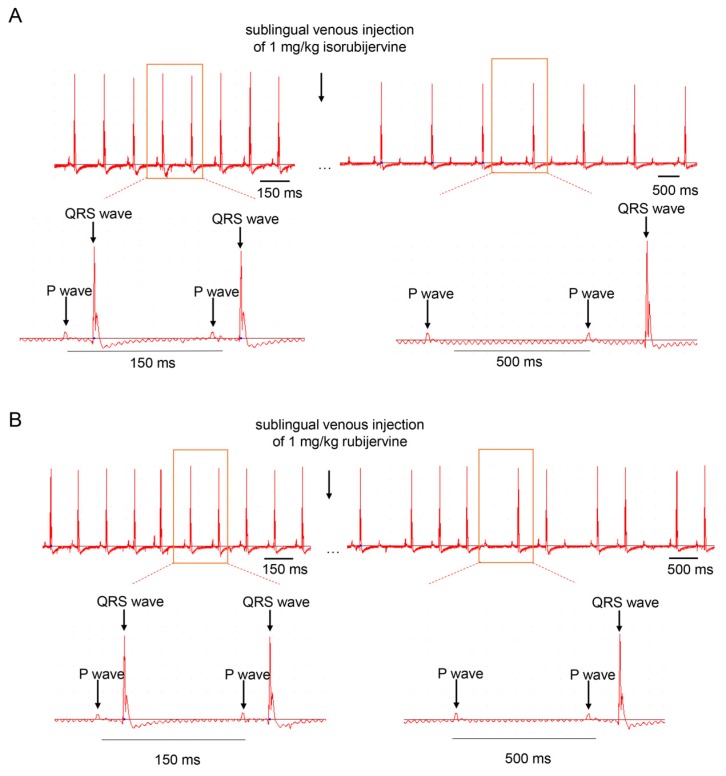
Representative ECGs of untreated, isorubijervine- and rubijervine-treated rats. Rats were treated with a sublingual venous injection of 1 mg/per kilogram of body weight. Both isorubijervine (**A**) and rubijervine (**B**) induced a periodic disappearance of the QRS wave (*n* = 3).

The QRS waves disappeared periodically after the sublingual venous injection of the two compounds, but the P wave was consistent (Figure 2). The two solanidine-type compounds induced atrioventricular conduction block *in vivo*.

### 2.3. Effects of Isorubijervine and Rubijervine on Macaque Blood Pressure

**Figure 3:**
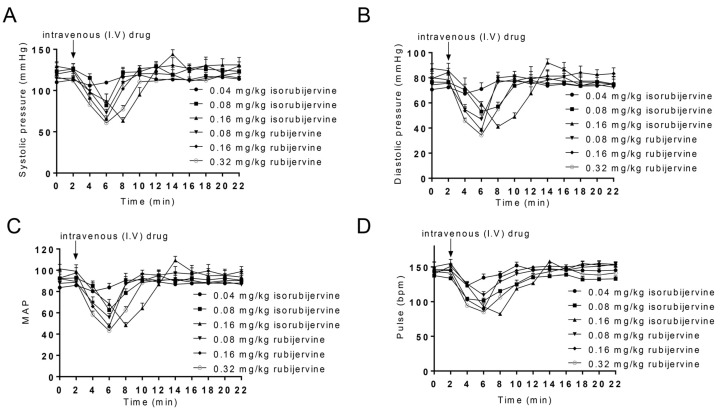
Responses to isorubijervine- and rubijervine-induced reductions in blood pressure in macaques. Isorubijervine- and rubijervine-induced reductions in systolic pressure (**A**); diastolic pressure; (**B**) mean arterial pressure (MAP) (**C**) and pulse (**D**). The duration of this effect was 10 min after intravenous injection of isorubijervine and rubijervine and was dose dependent (*n* = 3).

Different LD_50_ values between isorubijervine (1.14 mg/kg) and rubijervine (1.77 mg/kg) were observed in mice. Different VA concentrations were used to test the effect on blood pressure in macaques (Figure 3).

Intravenous (i.v.) isorubijervine concentrations were 0.04, 0.08 and 0.16 mg/kg and 0.08, 0.16 and 0.32 mg/kg for rubijervine. The systolic and diastolic pressures (SP and DP) and heart rate per min were recorded. Figure 3 shows that the heart rates of macaques treated with 0.04, 0.08 and 0.16 mg/kg isorubijervine were 123.00 ± 3.75, 101.33 ± 3.09 and 82.23 ± 1.24 beats/min, respectively, compared to the heart rate of 144.41 ± 7.27 beats/min in control macaques. The heart rates of macaques treated with 0.08, 0.16 and 0.32 mg/kg rubijervine were reduced to 109.33 ± 4.11, 89.23 ± 6.37 and 85.00 ± 3.55 beats/min, respectively, 4–6 min after i.v. injection.

The injection of 0.04, 0.08 and 0.16 mg/kg isorubijervine decreased SP, DP and mean arterial pressure (MAP) compared to control macaques without treatment. SPs decreased from 119.05 ± 8.77 mmHg to 106.03 ± 6.16, 82.00 ± 4.54 and 63.33 ± 3.29 mmHg approximately 4 min after the i.v. administration of 0.04, 0.08 and 0.16 mg/kg isorubijervine, respectively, and returned to control levels over the following 6 min. DP and MAP exhibited similar changes after isorubijervine injection. The DPs decreased from 78.4 ± 6.38 mmHg to 67.33 ± 1.69, 53.00 ± 3.26 and 41.00 ± 1.41 mmHg after the i.v. administration of 0.04, 0.08 and 0.16 mg/kg isorubijervine, respectively, and returned to control levels over the next 6 min. The MAPs declined from a control value of 91.96 ± 6.74 mmHg to 80.22 ± 3.17, 62.16 ± 3.68 and 48.44 ± 2.00 mmHg after the i.v. administration of 0.04, 0.08 and 0.16 mg/kg isorubijervine, respectively, and subsequently recovered to control levels in approximately 6 min.

SP, DP and MAP decreased in macaques after injections of 0.08, 0.16 and 0.32 mg/kg rubijervine. SPs decreased from the control level to 73.00 ± 3.56, 65.33 ± 3.29 and 60.66 ± 2.50 mmHg, approximately 4 min after the i.v. administration of 0.08, 0.16 and 0.32 mg/kg rubijervine, respectively, and returned to control levels over the next 6 min. DPs decreased from a control level to 47.00 ± 2.82, 38.33 ± 1.25 and 34.33 ± 2.49 mmHg, respectively, and returned to control levels over the next 6 min. MAPs decreased from a control level to 55.66 ± 3.06, 47.33 ± 1.90 and 43.11 ± 2.45 mmHg, respectively, and returned to control levels over the next 6 min. Isorubijervine and rubijervine induced transient bradycardia and hypotension, but the BP and heart rate quickly returned to normal.

### 2.4. Selective Activity of Isorubijervine and Rubijervine on Na_V_ Channel Subtypes

**Figure 4:**
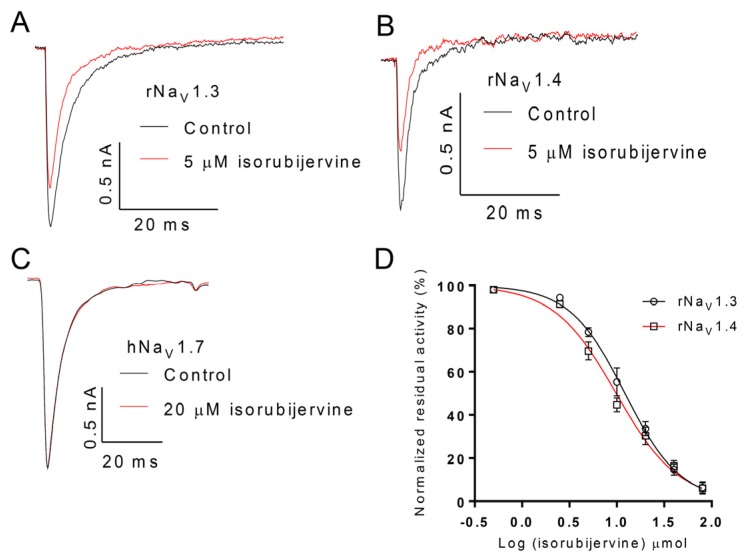
Effect of isorubijervine on rNa_V_1.3, rNa_V_1.4 and hNa_V_1.7 channels expressed in HEK293t cells. Control currents are shown in black, and the inhibition of rNa_V_1.3 (**A**); rNa_V_1.4 (**B**) and hNa_V_1.7 (**C**) by the indicated concentrations of isorubijervine are shown in red; (**D**) concentration-response curves for the inhibition of rNa_V_1.3, and rNa_V_1.4 channels by isorubijervine (*n* = 4).

**Figure 5:**
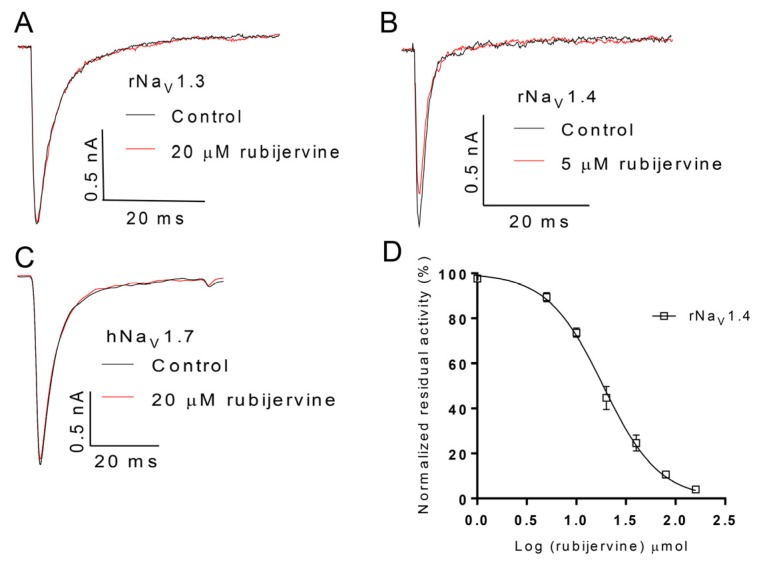
Effect of rubijervine on rNa_V_1.3, rNa_V_1.4 and hNa_V_1.7 channels expressed in HEK293t cells. Control currents are shown in black, and the inhibition of rNa_V_1.3 (**A**); rNa_V_1.4 (**B**) and hNa_V_1.7 (**C**) channels by the indicated concentrations of rubijervine are shown in red; (**D**) concentration-response curves for the inhibition of rNa_V_1.4 channels by rubijervine (*n* = 4).

The selective activity of isorubijervine and rubijervine on human Na_V_ (hNa_V_) or rat Na_V_ (rNa_V_) channel subtypes 1.3, 1.4 and 1.7 was investigated (Figure 4 and Figure 5). Isorubijervine inhibited rNa_V_1.3 and rNa_V_1.4 with IC_50_ values of 12.17 ± 0.77 and 9.82 ± 0.84 µM (Figure 4D), respectively. However, no effects on hNa_V_1.7 were detected at concentrations of up to 20 µM. Rubijervine inhibited rNa_V_1.4 with an IC_50_ value of 18.65 ± 1.01 µM (Figure 5D), but had no effects on rNa_V_1.3 or hNa_V_1.7 at concentrations up to 20 µM (Table S1).

### 2.5. Effects of Isorubijervine and Rubijervine on Na_V_1.5

**Figure 6:**
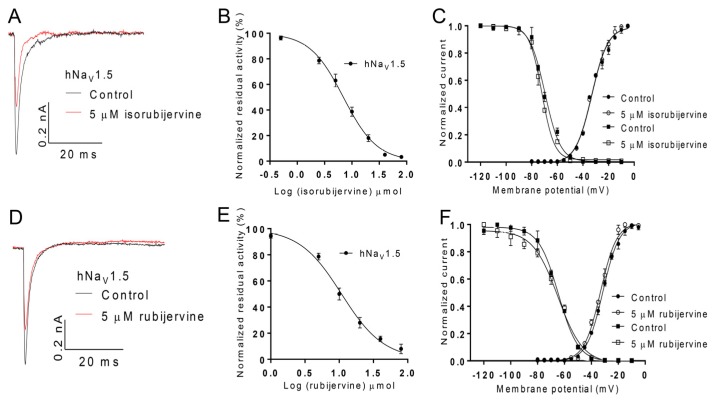
Effect of isorubijervine and rubijervine on hNa_V_1.5 channels expressed in HEK293t cells. (**A**) Inhibition of hNa_V_1.5 channel currents by 5 µM isorubijervine; (**B**) concentration-response curves for the inhibition of hNa_V_1.5 channels by isorubijervine (*n* = 4); (**C**) effect of 5 µM isorubijervine on the conductance–voltage (G–V) relationship (dot) and voltage dependence of steady-state inactivation (square); (**D**) inhibition of hNa_V_1.5 channel currents by 5 µM rubijervine; (**E**) concentration-response curves for the inhibition of hNa_V_1.5 channels by rubijervine (*n* = 4); (**F**) effect of rubijervine on the G–V relationship and voltage dependence of the steady-state inactivation (square) of hNa_V_1.5 channels (*n* = 5).

Additional attention was focused on the effects of isorubijervine and rubijervine on hNa_V_1.5 channels expressed in HEK293t cells because of the key role of hNa_V_1.5 in cardiovascular function and its specific distribution in the heart. Isorubijervine and rubijervine (5 µM) reduced the hNa_V_1.5 current by approximately 41% and 31%, respectively (Figure 6A,D). Isorubijervine and rubijervine inhibited hNa_V_1.5 currents in a dose-dependent manner, with IC_50_ values of 6.962 ± 0.422 and 10.81 ± 0.89 µM (Figure 6B,E), respectively. Isorubijervine (5 μM) did not induce a shift in the conductance–voltage (G–V) relationship or steady-state inactivation of hNa_V_1.5 (Figure 6C). Rubijervine (5 μM) only shifted the G–V relationship of hNa_V_1.5 approximately 2 mV in a negative direction, but it did not induce a shift in steady-state inactivation (Figure 6F).

## 3. Discussion

Alkaloids are an important active ingredient of *V. taliense* that have been used for medicinal purposes in China and Europe during the Middle Ages for the treatment of hypertension, stroke, excessive phlegm and epilepsy [8]. *V. taliense* also exhibits well-known poisonous characteristics, and the total alkaloids and veratramine of *V. nigrum* cause bradycardia, hypotension and even have led to deaths in Yunnan province [9,10]. VGSCs (Na_V_1.1–1.9) are expressed in different organs and tissues and exert different functions. For example, Na_V_1.3 is distributed in the central nervous system (CNS) and peripheral nervous system (PNS). Na_V_1.4 is distributed in skeletal muscle. Na_V_1.5 is only expressed in cardiac muscle. The VAs from *V. taliense* produce toxicities by acting on VGSCs, but the identity of the sodium channel subtypes that are the targets of the VAs and how the VAs affect sodium channels are not known.

Figure 6 shows that isorubijervine and rubijervine isolated from *V. taliense* potentially inhibited the Na_V_1.5 channel subtype with IC_50_ values of 6.962 and 10.81 µM, but these Vas exhibited no effects on K^+^ and Ca^2+^ channels (Figure S1). Na_V_1.5 is a cardiac sodium channel that plays a key role in the excitability of atrial and ventricular cardiomyocytes and rapid impulse propagation. These channels also conduct the fast inward Na^+^ current that initiates the depolarizing phase 0 of the cardiac action potential [11,12].

Obvious bradycardia symptoms, such as slow heart rate, were observed following isorubijervine and rubijervine administration in rats (Table 2). Decreased sodium channel availability or function may cause bradycardia [13]. Severe bradycardia due to the pro-arrhythmic effects of sodium channel blockers was reported [14]. Mutations in the gene encoding the cardiac sodium channel (SCN5A, Na_V_1.5) may contribute to bradycardia and sinus node dysfunction [15]. The effects of isorubijervine or rubijervine on Na_V_1.5 may be responsible for bradycardia.

Prolonged P wave duration was observed after isorubijervine and rubijervine treatment (Table 2). Over-expression of the SCN5A gene leads to a shorter P wave duration and PR interval in transgenic mice ECG [16], which suggests that Na_V_1.5 interference or blockade caused prolonged P wave duration, as observed in this report.

Isorubijervine and rubijervine treatment shortened the QT interval in rats (Figure 2), which is the same as other sodium channel blockers. For example, acute oral testing with the sodium channel blocker mexiletine shortened the QT interval in Long-QT (LQT) 3 patients [17]. The shortened QT possibly resulted from the reduction of action potential prolongation that was produced by delayed Na_V_1.5 inactivation.

Isorubijervine and rubijervine treatment in rats increased QRS amplitude, which is a symptom of cardiac hypertrophy. A reduction of Na_V_1.5 expression in cardiac hypertrophy mice was observed [18], which suggests that the downregulation of Na_V_1.5 expression or function is related to cardiac hypertrophy and increased QRS amplitude. The isorubijervine- and rubijervine-induced increases in QRS amplitude in the present study may be the result of Na_V_1.5 channel inhibition (Figure 6).

Hypotension was also observed in macaques after isorubijervine or rubijervine treatment (Figure 3), which was likely related to the effects of Vas on cardiac Na_V_1.5. Blockade of sodium channels, such as Na_V_1.3 in the peripheral nervous system, induces hypotension [19]. Therefore, isorubijervine- and rubijervine-induced hypotension may have resulted from the inhibition of Na_V_1.3 and/or Na_V_1.5.

## 4. Conclusions

This report focused on the effects of isorubijervine and rubijervine on cardiac Na_V_1.5 channels to reveal the possible mechanisms of cardiovascular toxicity. Notably, these two VAs also exhibited similar inhibitory abilities on two other sodium channels (Na_V_1.3 and 1.4). Na_V_1.3 is distributed in the CNS and PNS, and Na_V_1.4 is distributed in the skeletal muscle. The effects of isorubijervine and rubijervine on Na_V_1.3 and 1.4 channels may be related to their analgesic effect and skeletal muscle toxicity, respectively, which will be investigated in future studies.

## 5. Experimental Section

### 5.1. Plant Material

The roots and rhizomes of *Veratrum taliense* were collected from Dali in the Yunnan Province of China in July 2013. Dr. Yaping Liu Kunming from the Institute of Botany at the Chinese Academy of Sciences identified the plants. A voucher specimen (No. Liu 20130708) was deposited in the State Key Laboratory of Phytochemistry and Plant Resources in West China, Kunming Institute of Botany, Chinese Academy of Sciences.

### 5.2. Extraction and Isolation

The air-dried and powdered roots and rhizomes (10 kg) of *V. taliense* were extracted exhaustively using MeOH at room temperature. The combined MeOH extracts were concentrated and acidified with 0.5% hydrochloric acid. Extracts were filtered and partitioned with EtOAc to yield a non-alkaloid portion. The acid aqueous moiety was subsequently basified with 0.5% aqueous ammonia to pH 9–10 and partitioned with EtOAc to yield the alkaloid portion (400 g). The alkaloid fraction was subjected to silica gel column chromatography (CC) and eluted with CHCl_3_-MeOH (20:1, 15:1, 10:1 and 5:1, *v*/*v*) to yield four fractions (Fr. 1–Fr. 4). Fr. 2 (82 g) was subjected to CC on an RP-18 gel (medium pressure liquid chromatography (MPLC), MeOH-H_2_O, 60%–90%, *v*/*v*) to yield rubijervine (1.5 g) and isorubijervine (47 mg), isorubijervine (Figures S2–S4) and rubijervine (Figures S5–S7) are identified by NMR and ESI-MS. 

### 5.3. General Experimental Procedures

The 1D spectra were run on a Bruker AVANCE III–600 MHz (Bruker, Billerica, MA, USA), Bruker DRX-500 MHz spectrometer (Bruker, Billerica, MA, USA) or AV-400 MHz spectrometer (Bruker, Billerica, MA, USA), using tetra-methylsilane as an internal standard. Chemical shifts (δ) are expressed in ppm with reference to solvent signals. CC was performed on Silica gel (200–300 mesh, Qingdao Marine Chemical Ltd., Qingdao, China), RP-18 gel (20–45 µm, Fuji Silysia Chemical Ltd., Kasugai, Aichi Prefecture, Japan) and Sephadex LH-20 (Pharmacia Fine Chemical Co., Ltd., Uppsala, Sweden). Fractions were monitored using TLC (GF 254, Qingdao Haiyang Chemical Co., Ltd. Qingdao, China), and spots were visualized using Dragendorff's reagent. MPLC using a Buchi pump system coupled with a C18-silica gel-packed glass column (15 × 230 mm and 26 × 460 mm) was used.

### 5.4. Acute Toxicity Study

Healthy SD mice weighing 19–21 g were randomized into different groups to investigate the acute toxicity of isorubijervine and rubijervine. Two samples were prepared to the desired concentrations using menstruum (40% DMSO with normal saline). A 100-µL solution with different concentrations was administered to each mouse *via* tail vein injection. General health and mortality were monitored daily following treatment. The median lethal dose (LD_50_) was calculated based on animal death at different doses.

### 5.5. Surface ECG Measurements

Three-month-old rats were sedated via intraperitoneal injection of pelltobarbitalum natricum (2 g/100 mL saline solution, 0.3 mL/100 g body wt). The animals were placed in a supine position, and three limbs (two front paws, left leg) were attached to gel-covered silver wire loops. A three-lead surface ECG was recorded. The signals were pre-amplified and displayed on a computer (ECG parameters gain 1 mV, high-pass filtering 0.1 s, low-pass filtering 100 Hz). ECG data were acquired and analyzed using BL-420F (Taimeng, Chengdu, Sichuan, China) preamp and TM wave MFC Application 1.0 (Taimeng) software (Taimeng, Chengdu, Sichuan, China, 2006).

### 5.6. Measurement of Hemodynamic Responses

A total of 18 healthy male macaques, aged 2–3 years and weighing 3.5–4.5 kg, were randomly selected for experiments. Macaques were fixed on a table in the prone position. SP, DP and heart rates were recorded in one-minute segments using automated devices (YE660D, Yuwell, Nanjing, Jiangsu, China) under anesthesia (intramuscular injection of 20 mg/kg ketamine hydrochloride). The mean arterial pressure (MAP) was calculated using the following formula: MAP = DP + 1/3(SP − DP).

### 5.7. Patch-Clamp Recording of Sodium Channel Subtypes

Sodium channel subtypes (rNa_V_1.3, rNa_V_1.4, hNa_V_1.5 and hNa_V_1.7), β1 and eGFP were co-transiently transfected into HEK293t cells according to the manufacturer’s instructions (lipo2000, Invitrogen, Carisbad, CA, USA). The bath solution contained the following components: 140 mM NaCl, 3 mM KCl, 1 mM MgCl_2_, 1 mM CaCl_2_ and 10 mM HEPES, pH 7.3. The internal solution contained the following components: 10 mM NaCl, 3 mM KCl, 140 mM CsF, 1 mM, EGTA and 10 mM MgCl_2_, pH 7.3. Experimental data were acquired and analyzed using Clampfit 10.0 (Molecular Devices, Sunnyvale, CA, USA) and SigmaPlot (9.0, Systat Software Inc., San Jose, CA, USA, 2004) software.

### 5.8. Ethics Statement

The Animal Care and Use Committee of The Kunming Institute of Zoology, Chinese Academy of Sciences, reviewed and approved all of the procedures used in this study (2014-107). All experiments were performed according to good practices of laboratory animal management.

## Supplementary Materials

The following are available online at www.mdpi.com/2072-6651/8/1/12/s1.
