# Supplementary material for: Alkaloids from Veratrum taliense Exert Cardiovascular Toxic Effects via Cardiac Sodium Channel Subtype 1.5
Source: Toxins (Basel). 2015 Dec 30;8(1):12. doi: 10.3390/toxins8010012 (PMC4728534; doi:10.3390/toxins8010012)
Supplement: Supplementary file 1 [file toxins-08-00012-s001.pdf]

Supplementary Materials: Alkaloids from *Veratrum taliense* Exert Cardiovascular Toxic Effects via Cardiac Sodium Channel Subtype 1.5

Gan Wang, Ming-Qiang Rong, Qiong Li, Ya-Ping Liu, Cheng-Bo Long, Ping Meng, Hui-Ming Yao, Ren Lai and Xiao-Dong Luo

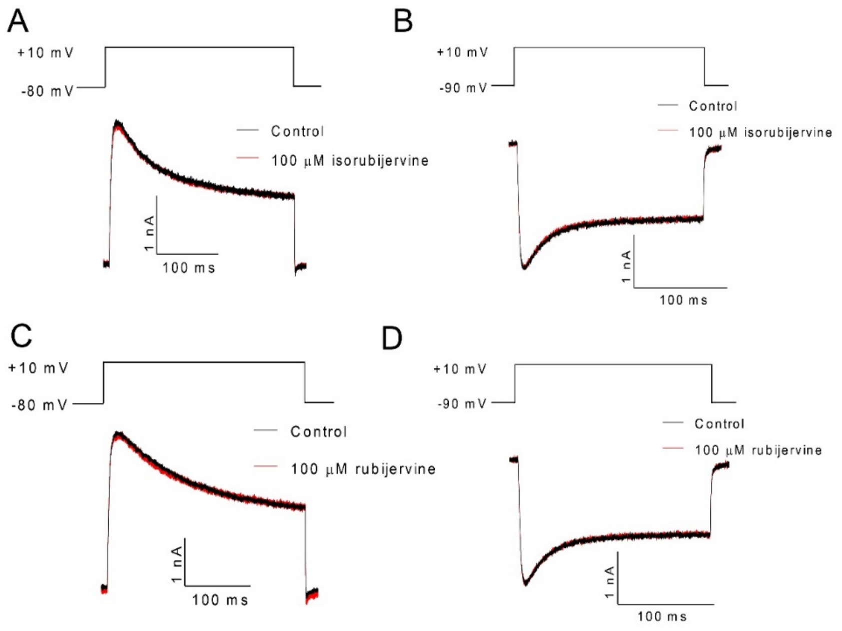

Figure S1. Effects of isorubijervine and rubijervine on dorsal root ganglion (DRG) neurons. Cells were evoked by a 500-ms depolarizing potential of +10 mV from a holding potential of −80 mV to record Kv currents. Cells were activated by a 150-ms step depolarization to +10 mV from a holding potential of −90 mV for Cav currents. The effects of isorubijervine (100 μM) on DRG Kv channel currents (**A**) and Cav channel currents (**B**). Effects of 100 μM rubijervine on DRG Kv channel currents (**C**) and Cav channel currents (**D**).

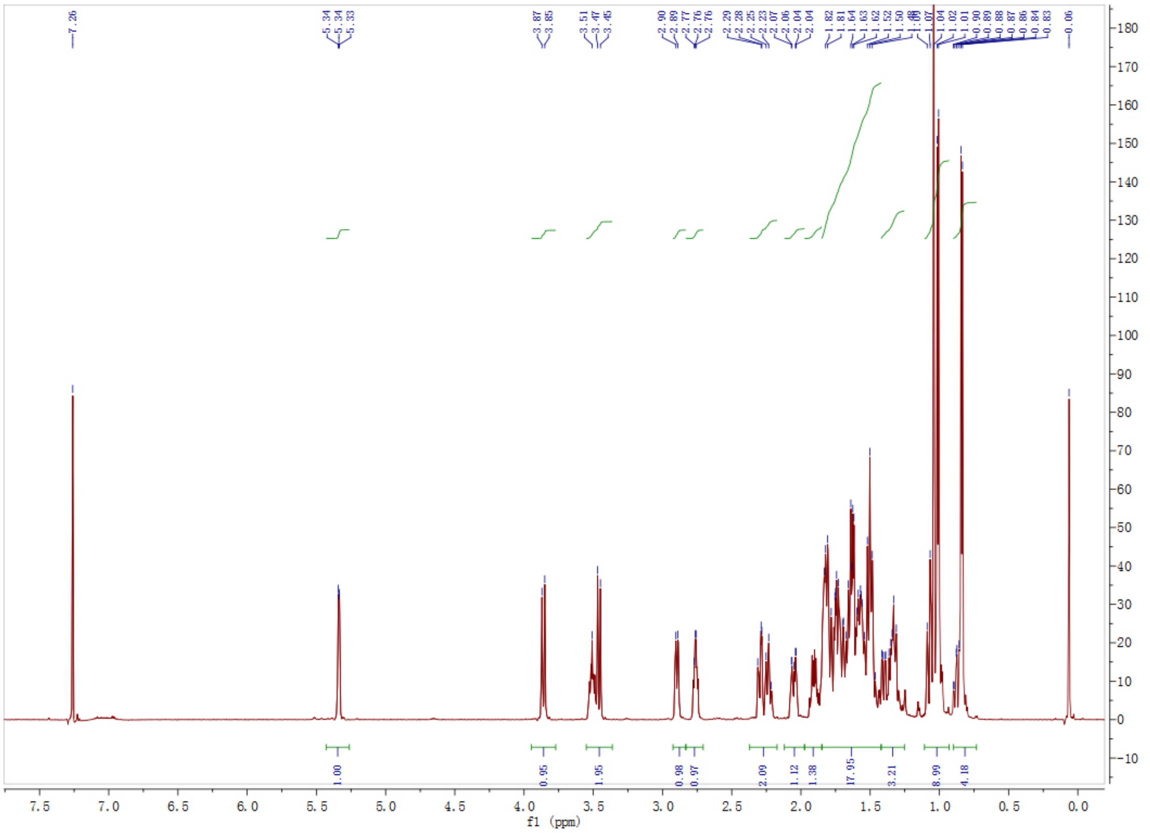

Figure S2. ^{1}H-NMR spectrum of isorubijervine in $CDCl_3$.

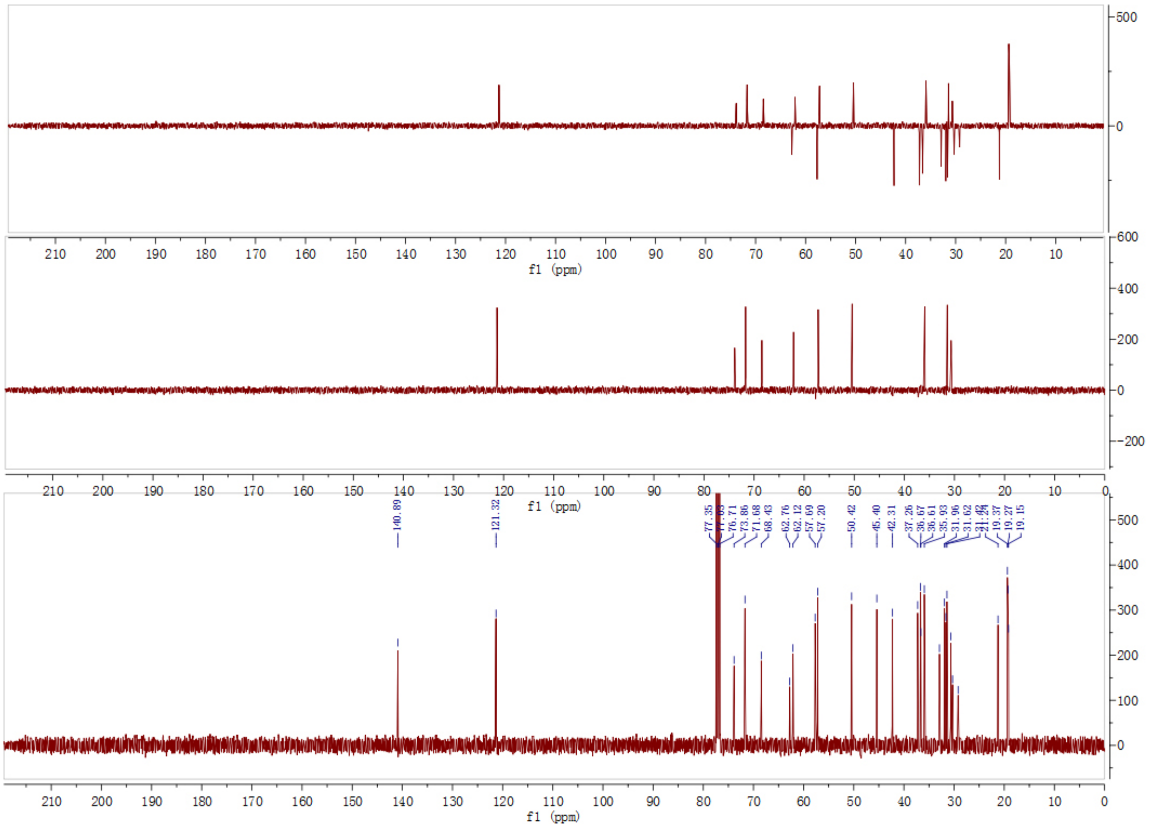

Figure S3. ^{13}C-NMR spectrum of isorubijervine in $CDCl_3$.

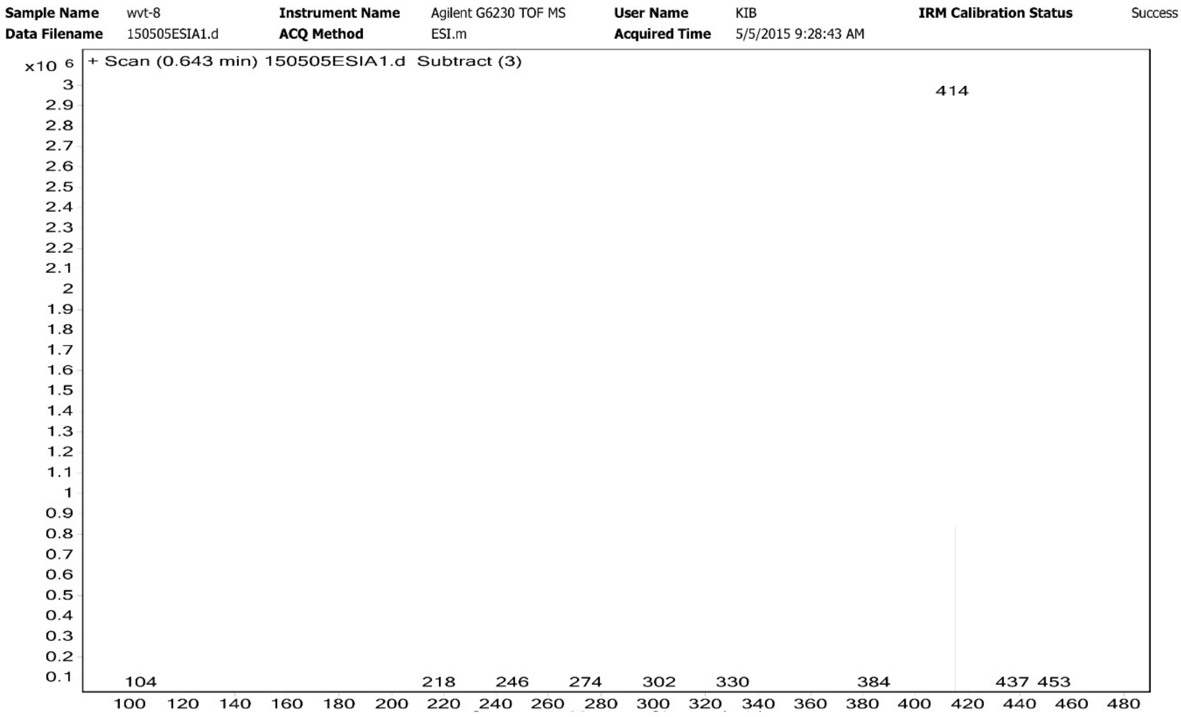

Figure S4. ESI-MS spectrum of isorubijervine.

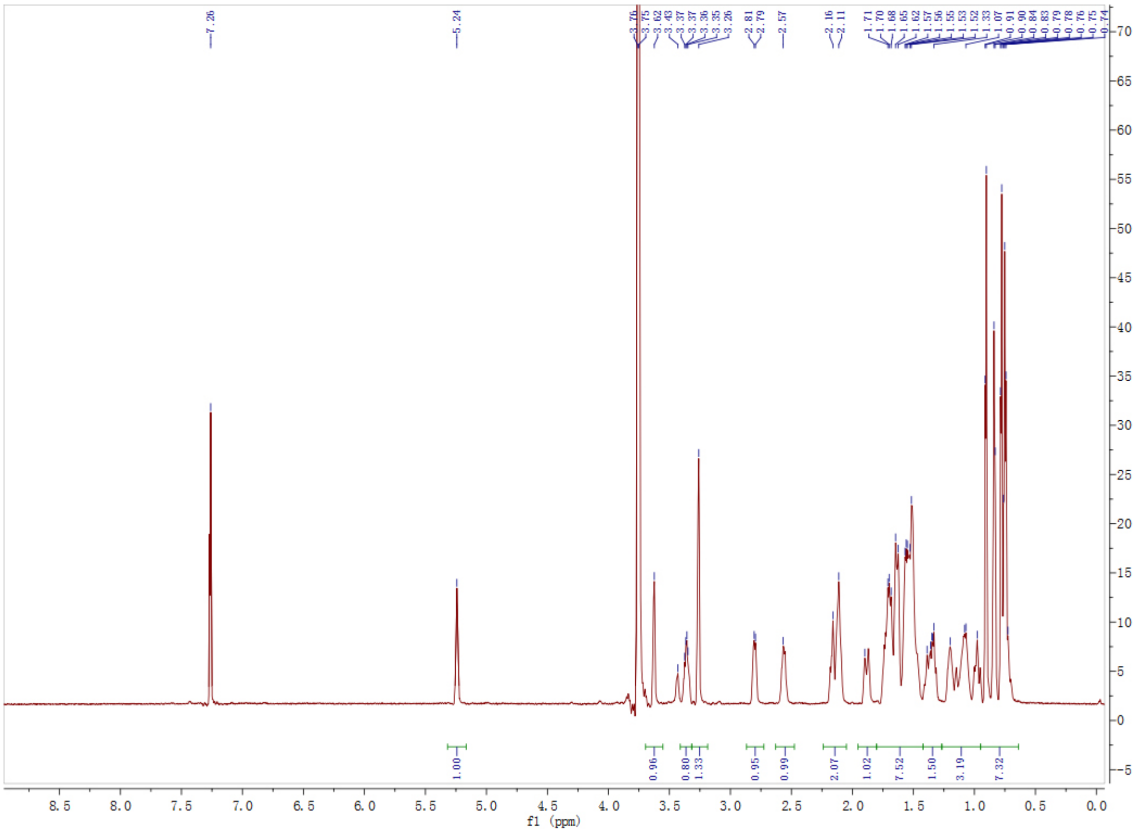

Figure S5. ^{1}H NMR spectrum of rubijervine in $CDCl_3$.

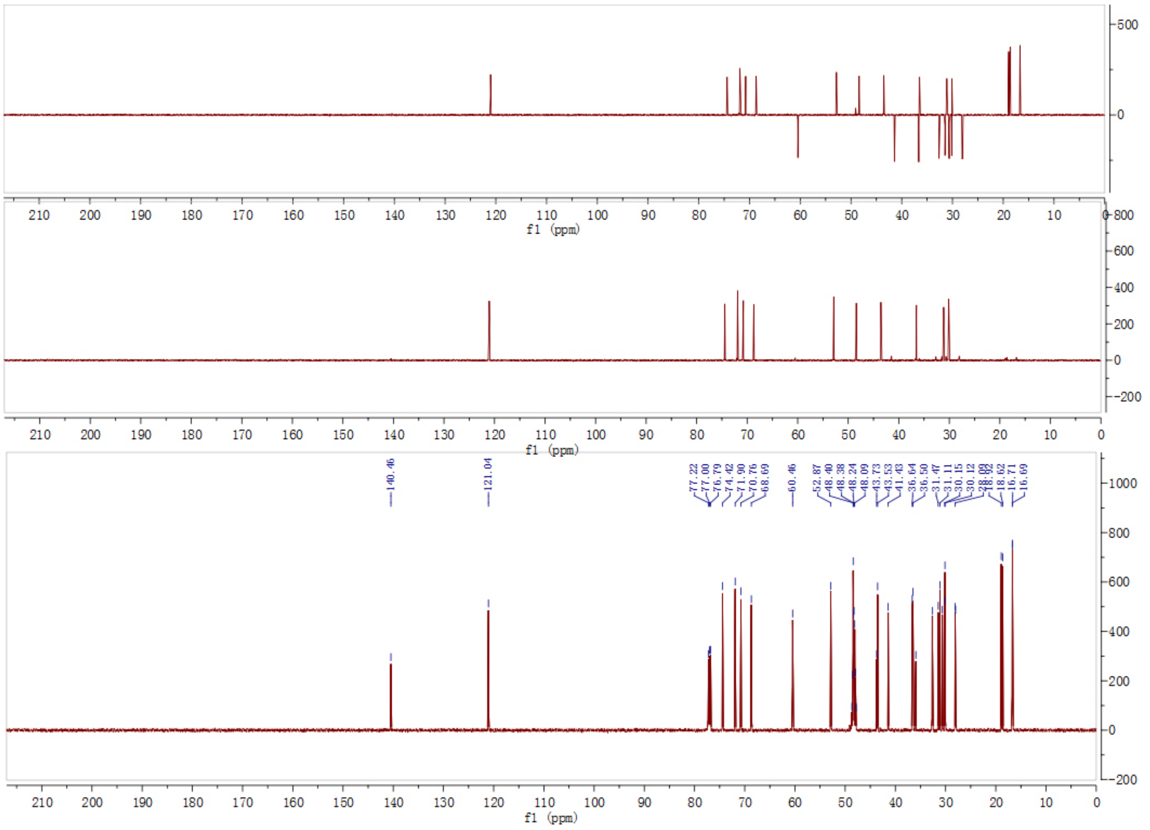

Figure S6. ^{13}C NMR spectrum of rubijervine in $CDCl_3$.

User Spectra

Fragmentor Voltage	Collision Energy	Ionization Mode
200	0	ESI

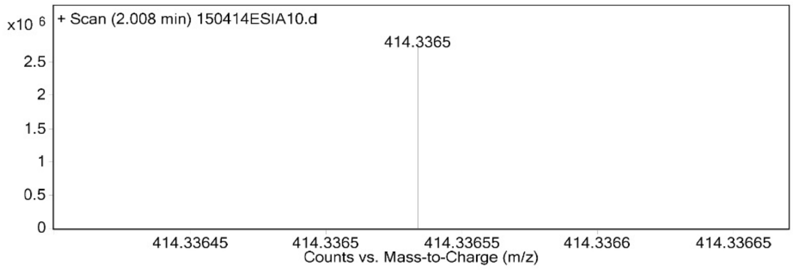

Peak List

m/z	z	Abund	Formula	Ion
121.0509	1	11,5048.14		
412.3221	1	5,9188.88		
414.3365	1	278,6094	C27 H44 N O2	M+
415.3409	1	81,8768.5	C27 H44 N O2	M+
416.3438	1	12,5858.18	C27 H44 N O2	M+
863.6427	1	6,4625.06		
922.0098	1	16,5486.72		
941.6582	1	6,8858.32		
1050.6346	1	9,9226.41		
1051.6375	1	6,1494.29		

Formula Calculator Element Limits

Element	Min	Max
C	0	200
H	0	400
O	0	6
N	1	1

Formula Calculator Results

Formula	CalculatedMass	CalculatedMz	Mz	Diff. (mDa)	Diff. (ppm)	DBE
C27 H44 N O2	414.3372	414.3367	414.3365	-0.1	-0.3	6.5000

--- End Of Report ---

Figure S7. high resolution electrospray ionization mass spectroscopy (HRESIMS) spectrum of rubijervine. DBE: double bond equivalents.

Table S1. IC_{50} of two compound on sodium channel (μM).

Compound	rNav1.3	rNav1.4	hNav1.5	hNav1.7
Isorubijervine	12.17 ± 0.77	9.82 ± 0.84	6.962 ± 0.422	-
Rubijervine	-	18.65 ± 1.01	10.81 ± 0.89	-

Notes: values are given as the mean ± SE, n = 4; no inhibitory activity detected at a concentration up to 20 μM.
